# Iron at the Interface of Hepatocellular Carcinoma

**DOI:** 10.3390/ijms22084097

**Published:** 2021-04-15

**Authors:** Rossana Paganoni, André Lechel, Maja Vujic Spasic

**Affiliations:** 1 Institute of Comparative Molecular Endocrinology, Ulm University, 89081 Ulm, Germany; rossana.paganoni@uni-ulm.de; 2 Department of Internal Medicine I, University Hospital Ulm, 89081 Ulm, Germany; andre.lechel@uni-ulm.de

**Keywords:** iron, hepcidin, ROS, hepatocellular carcinoma, hemochromatosis, HFE, NAFLD, metabolic syndrome

## Abstract

Cancer incidence and mortality are rapidly growing, with liver cancer being the sixth most diagnosed cancer worldwide and the third leading cause of cancer death in 2020. A number of risk factors have been identified that trigger the progression to hepatocellular carcinoma. In this review, we focus on iron as a potential risk factor for liver carcinogenesis. Molecules involved in the regulation of iron metabolism are often upregulated in cancer cells, in order to provide a supply of this essential trace element for all stages of tumor development, survival, proliferation, and metastasis. Thus, cellular and systemic iron levels must be tightly regulated to prevent or delay liver cancer progression. Disorders associated with dysregulated iron metabolism are characterized with increased susceptibility to hepatocellular carcinoma. This review discusses the association of iron with metabolic disorders such as hereditary hemochromatosis, non-alcoholic fatty liver disease, obesity, and type 2 diabetes, in the background of hepatocellular carcinoma.

## 1. Global Cancer Incidence

Cancer incidence rates and cancer mortality are rapidly growing worldwide. The number of new cases in the last 20 years has almost doubled, from 10.9 million cases in 2002 to 19.3 million in 2020, and cases are expected to rise in the future [1,2]. This trend is also observed within cancer mortality, with the death toll rising from 6.7 million in 2002, to 10 million in 2020 [1,2]. Global analysis of the incidence and mortality of various cancer types in both genders placed lung cancer as one of the most lethal, followed by female breast cancer, colorectal cancer, prostate cancer, stomach cancer, and liver cancer [1]. The prevalence and distribution of the main risk factors for cancer, several of which are associated with ageing, socioeconomic development and lifestyle, reproductive system, dietary choices, and metabolic and hormonal status, contribute to differing cancer profiles between world regions [3]. For instance, the incidence rate of lung cancers in men living in western countries is 39% in comparison to 10.3% in developing countries [1]. On the other hand, infection-associated cancers, such as cervical cancer, show the opposite ratio, with an incidence rate of 11.3% in the western world and 18.8% in developing countries [1].

## 2. Liver Cancer and Its Origin

Liver cancer is the sixth most diagnosed cancer type worldwide, and the third leading cause of cancer mortality in 2020 [1]. By incidence, China is ranked first, accounting for 46.7% of new cases of liver cancer last year, followed by the USA with 4.5% of new cases, with both being linked to an increased presence of metabolic disorders [1,4]. In twenty countries located predominantly in Sub-Saharan Africa and South-Eastern Asia, liver cancer is the leading cause of death due to its association with viral infections prevalent in these countries [5]. Liver cancer is relatively rare in Germany; however, it is one of the most common cancer-related deaths due to its high mortality rate and poor prognosis. In 2016, the incidence rate of liver cancer in Germany was approximately 9000 new cases per year and nearly 8000 deaths. The frequency of liver cancer in women is less than half of that in men, while the relative 5-year survival rate is 15% for both genders [6].

Liver cancer is a general denomination, and each form of cancer is named after the cell type that becomes cancerous. For example, hepatocellular carcinoma (HCC) and intrahepatic cholangiocarcinoma (ICC) are the most frequently occurring types of primary liver cancer, and together are among the most common cancers worldwide, with more than 900,000 new cases globally in 2020 [1]. In particular, HCC is the most common type of liver cancer, accounting for 75–85% of all liver cancers and resulting in more than 700,000 deaths/year worldwide [1]. It is the fifth most often diagnosed cancer in males, and the ninth most diagnosed in females [1]. HCC originates from the predominant type of liver parenchymal cells, hepatocytes, and usually develops within the presence of advanced chronic liver disease [5,7]. In contrast to HCC, ICC arises primarily from the epithelial lining of the bile duct (intra- and extra-hepatic bile duct) that carries bile to the gall bladder. The relative incidence of ICC is around 10–15% of all liver cancers [1]. The third most common primary malignancy of the liver is hepatic angiosarcoma (HA), that begins in endothelial cells of blood or lymphatic vessels [8]. HA is particularly rare, accounting for just 2% of liver cancers, and is characterized by a very rapid progression of the illness, with an average life expectancy of around 10 months after the first diagnosis [8].

## 3. Risk Factors for HCC

The type of liver damage development is dependent upon the nature and severity of the lesion. In general, liver carcinogenesis is a progressive multi-stage process that proceeds with chronic inflammation, followed by fibrosis, and a culmination into cirrhosis. Cirrhosis is the most advanced stage of liver fibrosis and is associated with a higher risk of malignant liver transformation into HCC. Major risk factors for HCC include viral infections such as hepatitis B and C, chronic alcohol consumption, and non-alcoholic fatty liver disease (NAFLD) [9,10]. Moreover, aflatoxin exposure, Wilson’s disease, glycogen storage disease, α1-trypsin deficiency, autoimmune hepatitis, obesity, diabetes, and primary biliary cirrhosis are further examples of conditions that induce chronic liver damage and thereby increase the susceptibility to HCC [11,12,13,14,15,16,17,18,19].

In this review, we particularly focus on iron as a risk factor for HCC.

## 4. Iron as a Risk Factor for Liver Carcinogenesis

### 4.1. Mechanisms of Iron-Mediated Carcinogenesis

Iron is one of the most common elements on our planet and an essential micronutrient for numerous biological processes. It is involved in a variety of fundamental cellular and biochemical processes, including oxygen transport, DNA synthesis, cell survival, energy metabolism, and cellular respiration due to its integration within heme- and iron-sulphur cluster-containing proteins [20,21,22]. Typically, cancer cells require iron during all stages of tumor development, survival, proliferation, and metastasis [23,24,25,26,27,28,29,30]. Molecules involved in the regulation of iron metabolism, such as transferrin receptor, ferritin H, and divalent metal transporter 1 (DMT1), are often upregulated in cancer cells in order to induce iron accumulation [31,32]. Observations of enhanced iron uptake and iron retention in cancer cells suggest that cancers are dependent on iron.

Various iron-driven mechanisms have been described to induce HCC, or cancer in general. The most important of these is the generation of reactive oxygen species (ROS) and the resulting oxidative stress. The hypothesis that oxidative stress is the underlying cause of liver damage in iron-excess conditions has emerged from the role of ‘free’ iron which is present when the iron-binding capacities of plasma transferrin or intracellular iron-storage molecule ferritin are surpassed. The redox-active properties of iron account for its toxicity exerted in “Fenton-type” reactions, where iron mediates conversion of generally inactive H_2_O_2_ into extremely reactive ·HO (Fe^2+^ + H_2_O_2_ → reactive intermediates → Fe^3+^ + HO + OH−) [33]. Superoxide anions can reduce Fe^3+^ back to Fe^2+^ (Fe^3+^ + O·^−2^ → Fe^2+^ + O_2_), thus allowing iron to act as a catalyst of the reaction. A transient pool of redox-active iron is present in the cytosol and mirrors the overall iron status of the cell. Pathological accumulation of iron within cells and tissues enhances generation of ROS and elicits toxic effects, reflected in the production of hydroxyl and lipid radicals, which attack proteins, nucleic acids, and carbohydrates [34]. In addition, ROS promotes carcinogenesis via mutagenesis, genomic instability, and DNA repair defects, thereby compromising cell viability and promoting cell death [35,36]. Besides the direct role of ROS in promoting carcinogenesis, ROS can serve as secondary messengers in intracellular signaling cascades, promoting and maintaining the oncogenic phenotype of cancer cells [37,38].

### 4.2. Role of Cellular Iron Metabolism in the Pathogenesis of HCC

Iron metabolism is a tightly controlled process at the cellular level. Cellular iron levels are regulated by F-box and leucine-rich repeat protein 5 (FBXL5), and iron regulatory protein 2 (IRP2) [39]. A recent publication from Nakayama’s lab described the importance of FBXL5-mediated cellular iron homeostasis in liver carcinogenesis [40]. Mice lacking *Fbxl5* are characterized as having excessive iron accumulation, resulting in subsequent oxidative stress, inflammation, and IRP2 accumulation. Exposure of *Fbxl5* knockout mice to the chemical carcinogen, diethylnitrosamine (DEN), or to virus-induced HCC, resulted in an increased mutational load and liver tumor formation [40]. Deletion of *Irp2* restored the lack of FBXL5 in the DEN model [40]. Moreover, low levels of *Fbxl5* expression in HCC patients were associated with a poor disease prognosis. This data highlights the importance of strict regulation of cellular iron levels, a major elicitor of oxidative stress, in protection against hepatocarcinogenesis.

### 4.3. Role of Systemic Iron Overload in the Pathogenesis of HCC

Likewise, iron metabolism is a precisely controlled process at the systemic level. The small hepatocyte-derived peptide hormone, hepcidin, synchronizes systemic iron fluxes to regulate adequate supplies of iron to tissues and cells, while preventing iron overload and iron-related toxicity [41,42,43]. Hepcidin acts by binding to the cellular iron exporter ferroportin (FPN) to trigger its internalization and degradation [44]. Hepcidin expression is regulated by several signals, including plasma iron levels, hepatic iron stores, inflammatory states, erythropoietic activity, and hypoxia [43].

Systemic iron overload, due to dietary, environmental, or genetic factors, can significantly promote tumorigenesis. In particular, excessive iron in the liver may act both directly and indirectly to induce carcinogenesis. One such direct role of excessive iron in the pathogenesis of HCC was confirmed in an animal model that was fed an iron-rich diet, which resulted in HCC developing in the absence of fibrosis and cirrhosis [45]. The findings by this study confirm a direct mutagenic and hepatocarcinogenic effect of free iron, elicited through the generation of ROS and oxidative damage [45]. The contribution of iron overload in cancer development is well-documented in patients suffering from a genetic iron overload disorder (hereditary hemochromatosis, HH), caused by mutations within the *HFE* gene. *HFE*-HH is characterized by excessive iron absorption from the diet and increased iron deposition in tissues [46,47,48]. Genetic data in mice and patients demonstrated a relative deficiency of iron-hormone hepcidin to be the hallmark of *HFE*-HH disorders [49,50,51,52,53,54]. Since there is no regulated mechanism to remove the excess of iron, progressive iron accumulation in various tissues occurs, which may consequently cause serious organ dysfunctions [46,55,56,57,58]. Excessive iron overload exerts a toxic effect on the liver and correlates with an increased risk of cirrhosis [59,60]. It has been widely reported that mutations in the *HFE* gene correlate with increased cancer risk [61], and that HH patients have a 20- to 200-fold higher probability to develop liver cancer compared to control patients, culminating in deaths in 45% of all HCC patients [59,62,63,64]. Moreover, a meta-analysis study identified a statistically significant association between the *HFE* H63D polymorphism and hepatocellular carcinoma, especially in Asian and African populations [65]. Interestingly, HCC has been documented to develop in iron-overload patients even in the context of non-fibrotic and non-cirrhotic livers, suggesting a more direct role of hepatic iron overload in triggering the malignant transformation of hepatocytes [66,67,68,69]. Of note, *HFE*-HH patients show higher rates of *p53* mutations (64–71%), compared to sporadic HCC patients, supporting a role of p53 in the hepatocarcinogenesis induced by iron overload [70,71]. *p53* is one of the most common onco-suppressor genes mutated in cancer [72]. The importance of p53 in tumor suppression is due to its ability to act as a transcription factor, and to regulate a series of target genes necessary for cell survival and death [73]. An earlier study by Hussain et al. demonstrated that increased oxidative stress (due to high levels of etheno-DNA adducts formed from oxyradical-induced lipid peroxidation) in non-tumorous human liver of *HFE*-HH patients, caused mutations in the *p53* tumor suppressor gene [74]. More recent studies showed that iron overload directly decreased p53 protein level and its activity in the liver, thereby facilitating hepatocarcinogenesis [75]. Consistently, *p53* expression is decreased in the liver of *Hfe*−/− mice and in wild-type mice fed with an iron-rich diet [75]. The molecular mechanism behind p53 vanishing has been ascertained in HepG2 cell lines: iron-containing porphyrin (heme) binds to p53 at the C-terminal end of the protein, thus interfering with p53-DNA interactions and triggering both the nuclear export and cytosolic degradation of p53 via proteasomes [75]. Additionally, p53 is involved in the regulation of key iron sensors such as iron-sulphur cluster assembly enzyme (ISCU) and hepcidin. More precisely, p53 controls the intracellular iron pool by specifically modulating IRP1 RNA binding activity via transcriptional regulation of the ISCU [76,77]. Furthermore, the promoter of hepcidin contains putative p53-responsive elements and thus can be activated by p53 and decreased when *p53* is silenced [78]. Therefore, following chronic iron overload, increased intracellular iron levels, the presence of oxidative stress, and reduced p53 activity may co-promote hepatocarcinogenesis.

Based on the above findings, it would be paramount to determine if *p53* expression is decreased in other forms of HH, such as those caused by the mutations in hepcidin, hemojuvelin, and ferroportin genes. These investigations may reveal whether low p53 levels may be considered a common denominator of HCC in HH conditions. Moreover, given that iron chelators such as desferrioxamine show an antitumor effect in patients with advanced HCC, it is crucial to investigate if iron chelation would result in the normalization of *p53* expression in the liver of HH-mice and patients [79].

## 5. Iron as a Co-Risk Factor in the Pathogenesis of HCC

Iron may also act synergistically with other risk factors in the pathogenesis of HCC, since chronic liver diseases such as chronic hepatitis, alcoholic liver disease, and NAFLD, associated with insulin resistance and the metabolic syndrome, are associated with iron overload.

### 5.1. Iron Overload and Viral Hepatitis

Hepatitis B virus (HBV) and hepatitis C virus (HCV) are major causes of viral hepatitis that lead to the development of cirrhosis and HCC [80,81]. The prevalence of HCC development in HBV carriers is up to 20-fold higher than the rate in non-carriers, as the DNA of HBV can integrate and replicate within cells, giving rise to chronic inflammation which is a predominant cause for fibrosis and cirrhosis [82,83]. It is estimated that 70–90% of HBV-associated HCC cases were identified in patients with cirrhotic liver [84]. Apart from random integration, HBV is able to disrupt cell cycle checkpoints and to induce chromosomal instability by protein HBx, thereby reinforcing the malignant transformation of hepatocytes. In addition to the prolonged HBV contagious period, HCV infection contributes as a major risk factor for liver cirrhosis [85]. Markedly, HCV-infected patients have an approximately 17-fold increased risk of developing HCC [86]. However, due to the RNA genome, the means by which HCV facilitates hepatocarcinogenesis differ greatly from mechanisms induced by HBV. So far, it is evident that structural and non-structural proteins of HCV are able to potentiate oncogenic transformation of hepatocytes by encouraging cell proliferation, DNA synthesis, steatosis, inflammatory processes, mitochondrial dysfunction, and insulin resistance, all of which lead to oxidative stress, genetic instability, and DNA damage; with cirrhosis and HCC as a potential outcome [87]. HCC risk drastically increases at the cirrhotic liver stage, suggesting a close association. The corresponding interplay of inflammatory responses, gene activation, and viral clearance suppression results in a conditioned environment that promotes cellular mutations leading to HCC.

HCV infection has been demonstrated to alter systemic and cellular iron homeostasis and its regulator hepcidin [88]. In the first phase of infection, hepcidin is more highly expressed in order to sustain the virus internalization though the iron importer, transferrin receptor 1 (TfR1) [89]. TfR1 was identified as a fundamental factor for HCV import in hepatocytes. Upon the binding of viral particles, TfR1 undergoes endocytosis, thus translocating viruses into intracellular compartments [89,90]. Indeed, TfR1 knockdown and antibody blocking inhibit HCV infection in Huh7 hepatoma cells [89]. In the following chronic phase, HCV infection leads to the downregulation of hepcidin in the plasma, with a resulting increase in iron absorption, as patient data clearly show [91,92]. Accordingly, these patients displayed increased transferrin saturation, raising the concentration of plasma ferritin and iron deposition in the liver [93]. Of interest is that the higher the iron levels are, the lower the response to the canonical therapy used for HCV infection is (interferon and ribavirin treatment) [94]. Moreover, the reduction in excess liver and body iron stores by phlebotomy was shown to ameliorate the course of hepatitis C and significantly improved the interferon effect [95,96].

Up to now, it is clear that HCV infection can induce iron overload in the liver and then behave as cofactors to worsen hepatic disease, characterized by advanced fibrosis, cirrhosis, and eventually even HCC. Yet, no data have demonstrated that iron increases susceptibility to HCV; quite the opposite, many studies now agree that the presence of HFE mutations alone does not significantly increase the risk of HCV disease [97].

### 5.2. Iron Overload and Chronic Alcoholic Liver Disease

Chronic alcohol consumption is a risk factor for the development of liver cancer, as demonstrated by its contribution to 30% of global HCC deaths [98,99,100,101]. Alcohol consumption increases the incidence of HCC in conjunction with other risk factors for chronic liver disease, especially viral hepatitis, diabetes, and obesity [102,103]. Chronic alcohol intake alters the liver architecture and compromises the functional capacity of the liver by triggering steatosis, steatohepatitis, and cirrhosis [104,105]. These pathological events are subsequently sustained and participate in the induction of carcinogenic processes. The mechanisms by which alcohol can mediate carcinogenesis are well known and were recently reviewed in [105]. Ethanol is first metabolized into acetaldehyde, which causes major cell damage by forming DNA and protein adducts [105]. In addition, alcohol increases gut permeability and the translocation of bacteria-derived lipopolysaccharide (LPS) from the gut to the liver, where the Kupffer cells start producing pro-inflammatory cytokines, triggering major biological pathways involved in hepatocarcinogenesis [106,107]. Moreover, the elevated production of cytochrome P450 2E1 (CYP2E1) and iron-induced ROS further aggravates the liver function by the impairment of antioxidant defenses and DNA repair mechanisms [108]. Hepatic iron accumulation and chronic alcohol consumption have long been associated together [109]. High liver iron was found to be predictive of HCC development or death in patients with alcoholic cirrhosis [110,111]. Both in vitro and in vivo models show that alcohol exposure directly downregulates hepcidin and simultaneously increases the expression of iron transporter proteins (DMT1 and FPN) in the duodenum, exacerbating the iron overload condition [112]. This effect of alcohol on hepcidin can be abolished with antioxidant treatment, pointing out the essential role of oxidative stress in the regulation of hepcidin expression [112]. In conclusion, alcohol abrogates the protective effect of hepcidin in iron overload by rendering the synthesis of hepcidin in the liver insensitive to body iron levels. Deregulation of hepcidin synthesis in the liver may be one of the underlying mechanisms by which alcohol consumption leads to iron overload.

### 5.3. Iron Overload as a Risk Factor in Progression of NAFLD to Non-Alcoholic Steatohepatitis (NASH), Cirrhosis and HCC

In several studies conducted in western countries, 30 to 40% of patients with HCC did not have chronic hepatitis infection, implying the presence of other causes of disease such as fatty liver disease, due to either alcohol abuse or NAFLD. A meta-analysis of 86 studies on 8.5 million subjects from 22 countries revealed that the global prevalence of NAFLD was 25.24% [113]. In particular, in the USA, 83.1 million people (~25% of the population) were diagnosed with NAFLD in 2015 and that number is expected to increase to 100.9 million by 2030 [114]. The pandemic of NAFLD represents the heaviest burden among modern liver diseases, not only due to its epidemiology, but also because of the risk of progression to cirrhosis and its associated complications, such as hepatic decompensation, HCC, and death. One study demonstrated that the standardized incidence ratio of HCC in patients with NAFLD was 4.4 after 16 years of follow-up [115]. The prevalence of NAFLD-related HCC is also rising worldwide and 4–22% of HCC cases in western countries are now attributed to NAFLD [115,116,117,118]. Meanwhile, in Asia, where viral hepatitis remains endemic, 1–2% of HCC cases are attributable to NAFLD [119,120]. Moreover, non-alcoholic fatty liver disease is present in up to 90% of all obese people and in up to 70% of people with type 2 diabetes (T2D), being a further possible risk factor for HCC [121,122,123,124].

NAFLD is a broad spectrum of liver diseases ranging from simple steatosis, such as the presence of fatty liver alone, to non-alcoholic steatohepatitis (NASH) and cirrhosis, that may progress to HCC [125,126]. A major hallmark of NAFLD is the accumulation of triglycerides in the cytoplasm of hepatocytes, that arises from an imbalance between lipid acquisition (i.e., fatty acid uptake and de novo lipogenesis) and lipid removal (i.e., mitochondrial fatty acid oxidation and export as a component of VLDL (very-low-density lipoprotein) particles [127,128].

Accumulating evidence suggests a link between altered iron metabolism and NAFLD. Iron has been implicated in the pathogenesis of NAFLD via oxidative stress. An excess of iron is the major cause of ROS production and oxidative stress has been demonstrated to be the starting point of the hepatic and extrahepatic damage in NAFLD [129,130]. Hepatic iron loading was shown to up-regulate cholesterol biosynthesis pathways and has been proposed as an additional mechanism contributing to iron-induced liver injury in fatty liver disease [131]. Concurrently, the presence of oxidative stress in the hepatocytes stimulates liver stellate cells to increase the formation of extracellular matrix components, which often causes fibrosis and cirrhosis and might lead to HCC development [19,47]. Last but not least, iron may also contribute to liver injury in NAFLD by generating endoplasmic reticulum (ER) stress. Tan et al. demonstrated that iron overload in conditions of alcohol- and obesity-induced liver injury significantly increased the extent of liver injury, steatohepatitis, and liver fibrosis through the induction of unfolded protein response and ER stress [132,133].

Progression from simple steatosis to NASH, which is a serious risk factor for cirrhosis and HCC, correlates with an increased concentration of iron in hepatocytes, increased production of lipid peroxides, organelle dysfunction, and eventually cell death [134,135]. In NASH, oxidative stress leads to cell death via depletion of ATP, NAD, and glutathione, and by direct damage to DNA, lipids, and proteins within hepatocytes [130]. Furthermore, oxidative stress leads to an increase in the production of pro-inflammatory cytokines and a fibrogenic response [130].

Iron accumulation in the liver is believed to be an aggravating factor. Elevated levels of serum ferritin, iron, and transferrin saturation, and an increase in hepatic iron staining were detected in more than 30% of patients with NAFLD and correlated with insulin resistance, advanced fibrosis, NASH, and an increased long-term risk of death [136,137,138,139]. Nelson et al. performed a study in which they divided NAFLD patients into three groups depending on iron localization in the liver cells (hepatocytes, endothelial cells, and Kupffer cells) [140]. The authors demonstrated that iron retention in endothelial cells and Kupffer cells aggravated NAFLD and increased fibrosis, inflammation, hepatocyte ballooning, and definite NASH compared to patients with iron deposition in hepatocytes and to those having mixed iron patterns [140]. Sorrentino et al. demonstrated that the hepatic iron was significantly higher in HCC-NASH patients than in HCC-free NASH controls, and, more importantly, in the former group the liver iron overload was mainly associated with Kupffer and sinusoidal cells [141]. Similarly, *HFE*-HH patients with NAFLD have more severe liver damage due to the iron deposition in hepatocytes and in non-parenchymal cells, and furthermore, these patients develop NASH even in the presence of less severe metabolic abnormalities, suggesting that iron overload may contribute to NAFLD onset and exacerbate the progression to NASH [142,143,144]. By contrast, hepatocellular iron excess in mouse models of hemochromatosis (such as *Hfe*−/−, *Hjv*−/−, and *Hfe*−/−/*Hjv*−/− double knock-out) did not aggravate diet-induced steatosis to steatohepatitis or early liver fibrosis [145]. Collectively, these studies reveal a still unresolved controversy in the field on whether iron excess in hepatocytes or Kupffer cells, or both, acts as a driver for progression of NAFLD to NASH and advanced liver disease.

Recently, Tsurusaki et al., showed that ferroptosis, a type of “programmed necrosis” described as an iron- and lipid hydroperoxide-dependent nonapoptotic cell death, may be the trigger for initiating inflammation in NAFLD and could be observed as the first event towards steatohepatitis [146,147]. Nonetheless, more investigations are needed to demonstrate the implication of ferroptosis in NASH and the possible effectiveness of ferroptosis inhibition, both in murine models and patients with NASH.

### 5.4. Iron as a Risk Factor in NAFLD Associated with Insulin Resistance and the Metabolic Syndrome

T2D has been associated with an augmented risk of developing different types of cancers, including liver cancers [148,149,150,151,152,153,154]. In a large cohort study, Inoue et al. showed that the risk of malignancy was 2.8-fold higher in patients with T2D than in those without [149]. Similar conclusions were drawn from case-control studies conducted in the population of the USA, where the proportion of HCC patients with diabetes was twice the controls, demonstrating that T2D increased the risk for HCC [150]. Although further evaluation is required to draw definitive conclusions, it is estimated that T2D can increase the risk of HCC by roughly two- to three-fold, and the development of HCC is the most worrisome liver-related complication in diabetic patients. Clinical observation studies demonstrated an increased incidence of T2D in patients with pathologic iron overload, including HH (13–22% prevalence), beta thalassemia (6–14% incidence), and Friedreich ataxia [155,156,157,158,159,160,161]. Importantly, even high levels of dietary iron or transfusional iron overload impart an increase in diabetes-related risks [162,163,164,165]. Iron overload in hepatocytes may induce insulin resistance via oxidative stress, increased accumulation of ROS, and subsequent impairment of signaling cascades such as phosphatidylinositol 3-kinase (PI-3K), adiponectin, interleukin, and TNF-α [166,167,168,169,170]. Placing obese mice or mice fed a high-fat diet on iron chelation therapy, or on an iron-deficient diet, resulted in significant protection from diabetes that was relayed by both increased insulin secretion and sensitivity [171]. Although the exact molecular link between iron and insulin resistance remains unknown, it is postulated that dysregulated expression of hepcidin and of molecules involved in cellular iron export may play a role. Interestingly, metformin, the first-line drug for T2D therapy, was recently shown to modulate iron metabolism by inhibiting bone morphogenetic protein 6 (BMP6)-induced hepcidin expression independently from protein kinase AMP-activated catalytic (AMPK) activation (canonical metformin activity) [172,173]. Metformin increases the orphan nuclear receptor SHP which interacts with SMAD1 (small mothers against decapentaplegic) proteins and suppresses BMP6-mediated recruitment of the SMAD1–SMAD4 complex to the hepcidin gene promoter [172,174]. Another proposed mechanism by which metformin regulates hepcidin levels is through inhibition of STAT3 activation by promoting the proteasome-mediated degradation of JAK2 [175]. Indeed, metformin pre-treatment significantly reduced the IL-6 response with a consequent reduction in pSTAT3 and hepcidin levels [175]. Moreover, metformin was associated with decreased serum hepcidin levels in Chinese T2D patients [175]. Thus, beneficial effects of metformin in inhibiting tumorigenesis could be, at least in part, ascribed to its ability to modulate hepcidin expression and, thus, the iron metabolism, since a reduction of 31% in cancer incidence was noted in diabetic patients undergoing metformin treatment [176]. Given the very high prevalence of the metabolic syndrome worldwide, and that even a small increase in risk related to obesity or diabetes could potentially translate into a large number of HCC cases, future investigations into the role of iron and its metabolism in the progression of HCC in the background of metabolic syndrome are urgently needed.

## 6. Conclusions

Iron overload, as it is observed in patients with hereditary hemochromatosis, is an important risk factor in liver carcinogenesis. The accumulation of iron in hepatocytes promotes oxidative stress, cell death, and compulsory liver regeneration. These factors lead to an accumulation of DNA damage, and subsequently to the malignant transformation of hepatocytes. There is experimental evidence that iron supports a carcinogenic or co-carcinogenic role in carcinogenesis. So far it is not clear if the accumulation of iron is a main driver for liver carcinogenesis, but it is definitely known that iron overload is an additional factor which will lead (in combination with disease models of liver cancer) to an increased mutational load and to tumor formation. Therefore, the modification of the cellular and systemic iron content might demonstrate an important factor for liver cancer prevention or a delay in liver cancer progression. Due to the limited understanding of the role of iron in liver cancer, the biology of iron metabolism, and the dysfunctional regulation of iron, it offers new challenges to unravel liver tumor pathogenesis and to develop new therapeutic strategies based on iron regulation.

## Data Availability

Not applicable.
